# Pulmonary Emboli Mimicking ST-Elevation Myocardial Infarction Patterns

**DOI:** 10.7759/cureus.72706

**Published:** 2024-10-30

**Authors:** Patrick Bruss, Ammar F Chauhdri, Ryan Gombash

**Affiliations:** 1 Emergency Medicine, ProMedica Monroe Emergency Medicine Residency, Monroe, USA; 2 Emergency Medicine, Wayne State University School of Medicine, Detroit, USA; 3 Emergency Medicine, ProMedica Monroe Regional Hospital, Monroe, USA; 4 Emergency Medicine, Emergency Physicians of Northwest Ohio (EPNO), Toledo, USA

**Keywords:** connexin-43 dysregulation, ct angiography, electrocardiogram (ecg), hypoxia, pulmonary embolism, right ventricular strain, st-elevation myocardial infarction

## Abstract

A pulmonary embolism (PE) is a serious condition characterized by obstruction of the pulmonary arteries, often presenting with symptoms such as sudden shortness of breath and chest pain. While pulmonary emboli and ST-segment elevation myocardial infarctions (STEMI) are distinct clinical entities, they can present with similar electrocardiogram (ECG) findings, complicating diagnosis and management. This study presents two case reports of patients who exhibited ECG changes resembling STEMI but were ultimately diagnosed with PE via CT angiography (CTA). The first case involved a 67-year-old female with a history of knee surgery and recent immobility, presenting with ST elevation in leads V1 and V2, whose CTA revealed bilateral segmental pulmonary emboli with right heart strain. The second case involved a 55-year-old female with COPD and recent surgery, presenting with ST elevation in lead aVR and V1, whose CTA also confirmed bilateral pulmonary emboli with right heart strain. The cases demonstrate the need for comprehensive clinical evaluation and advanced imaging to distinguish between PE and STEMI, especially in patients with risk factors for venous thromboembolism (VTE). We also aimed to explore potential mechanisms underlying the ECG similarities, including right ventricular strain and Connexin-43 (Cx43) dysregulation. The goal of the study is to highlight how an acute PE can mimic a STEMI and highlight the diagnostic challenge in a patient with this presentation.

## Introduction

Acute coronary syndrome (ACS) is a major cause of global morbidity and mortality with 805,000 Americans affected each year [1]. Venous thromboembolism (VTE) affects about one in 1,000 people yearly, with deep vein thrombosis (DVT) present in 33% of cases and pulmonary embolism (PE) found in 51.2-97% of DVT cases [2].* * A PE is a serious and potentially life-threatening condition that occurs when a blood clot obstructs the pulmonary arteries or their branches, often originating from a DVT in the lower extremities. This obstruction disrupts blood flow in the lungs and can lead to significant respiratory and cardiovascular complications. PE typically presents with symptoms such as sudden shortness of breath, chest pain, and, in severe cases, syncope or hemodynamic instability [3]. Risk factors for PE are closely associated with those for DVT and include both genetic and acquired factors. Genetic predispositions such as factor V Leiden mutation and prothrombin gene mutation increase the likelihood of clot formation. Genetic factors can be identified in up to 30% of patients with VTE, which is mainly attributable to factor 5 Leiden and prothrombin mutation. Antithrombin 3 protein C and protein S deficiencies are rare ( approximately 1% of the population) but are more associated with thrombosis [4]. Acquired risk factors encompass prolonged immobility, recent surgery, malignancy, obesity, pregnancy, and use of oral contraceptives. Other significant risk factors include fractures, heart failure, major trauma, and a history of VTE [3]. The treatment of PE primarily involves anticoagulation to prevent further clot formation and reduce mortality [3]. In hemodynamically stable patients, anticoagulants such as low-molecular-weight heparin, unfractionated heparin, or newer oral anticoagulants are commonly used. In cases of hemodynamic instability or massive PE, thrombolytic therapy, surgical embolectomy, or percutaneous catheter-directed therapy may be necessary to rapidly restore pulmonary blood flow and prevent right ventricular failure [3]. 

In contrast to PEs, an ST-segment elevation myocardial infarction (STEMI) is a severe form of ACS characterized by the presence of ST-segment elevation on an electrocardiogram (ECG), indicative of transmural myocardial ischemia. STEMIs are typically caused by an acute blockage of a coronary artery, leading to significant morbidity and mortality if not promptly treated [5]. The ECG findings in a STEMI are crucial for diagnosis and treatment planning, displaying hallmark features such as ST-segment elevation (often accompanied by reciprocal ST-segment depression in the opposing leads) in two or more contiguous leads, which reflect the area of myocardial injury [6]. * *The most current definition of ST-elevation myocardial infarction is as follows: new ST-segment elevation at the J-point in 2 contiguous leads, with a cutoff greater than 0.1 mV in all leads except V2 and V3. In leads V2 through V3, the cutoff is greater than 0.2 mV in men older than 40 years and greater than 0.25 mV in men younger than 40 years, or greater than 0.5 mV in women [6]. In addition to ST-segment elevation, other ECG changes can include the development of pathological Q-waves and T-wave inversions, which provide further diagnostic information and can help in assessing the extent and progression of myocardial damage [7]. The accurate interpretation of these ECG changes is essential for the early identification and management of a STEMI, enabling timely reperfusion therapies such as percutaneous coronary intervention (PCI) or thrombolysis, which are critical in reducing myocardial damage and improving patient outcomes [5]. 

PEs have been known to mimic other cardiovascular events on an ECG, including STEMIs, often appearing as ST-segment elevations in the right precordial leads [8]. This phenomenon, though rare, complicates the diagnosis and management of PE, necessitating heightened clinical awareness and prompt diagnostic measures [7,9]. The presentation of PE with ECG changes characteristic of STEMI, such as ST elevations in leads V1-V4, reinforces the importance of differentiating between these conditions to avoid inappropriate interventions like unwarranted coronary angiography [8]. Several case reports and studies have documented this diagnostic challenge, emphasizing the role of comprehensive clinical evaluation and imaging techniques like computed tomography pulmonary angiography (CTPA) for accurate diagnosis [10].

In this case report, we describe two individuals who presented to the emergency room (ER) with PEs that exhibited ECG changes resembling STEMIs. This unusual presentation underscores the need for thorough diagnostic evaluation to distinguish between these conditions and implement appropriate treatment strategies [7]. This report aims to highlight the clinical and diagnostic intricacies involved in diagnosing such cases, as well as a preliminary review of potential mechanisms as to why a PE may present itself as a STEMI.

## Case presentation

Case 1

**Figure 1:**
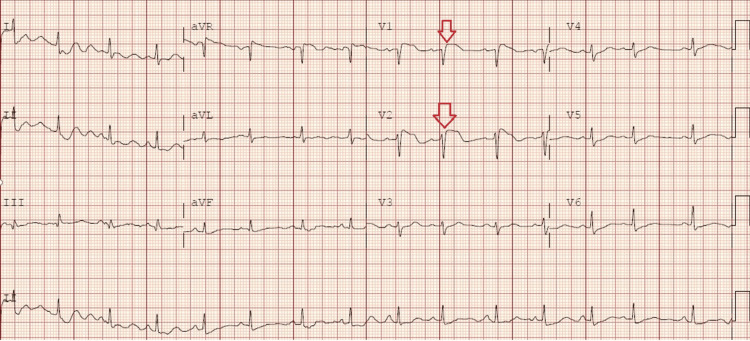
ECG on arrival to ED. New ST elevation in V1 and V2 (red arrows) ECG, electrocardiogram

**Figure 2:**
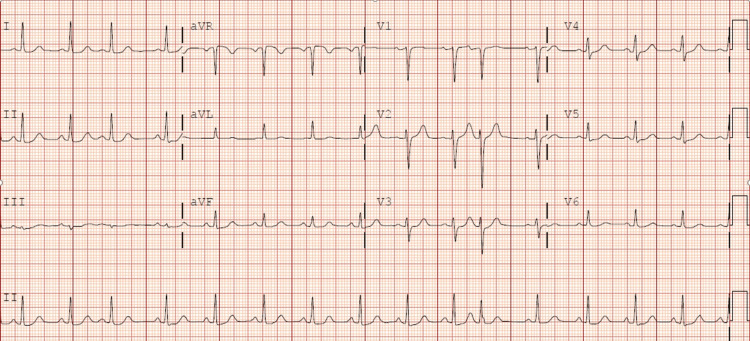
Baseline ECG from a prior visit. No evidence of ST elevations in V1 or V2 ECG, electrocardiogram

**Figure 3:**
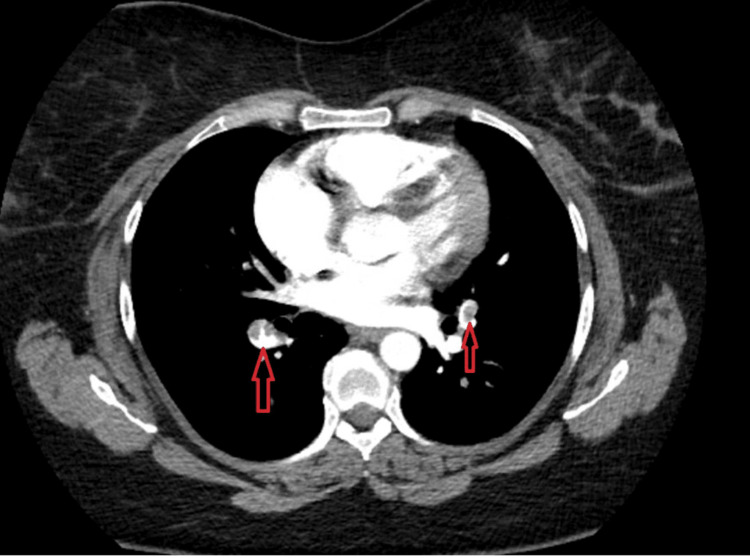
CT angiogram. Notable for bilateral segmental pulmonary emboli with (red arrows) evidence of right heart strain

A 67-year-old female presented to the ED complaining of shortness of breath and dizziness. She described a gradual onset of symptoms over the past 24 hours, which had worsened, prompting her to seek medical attention. Her medical history included sleep apnea and knee surgery two months prior, as well as a recent long car trip out of state. On examination, the patient appeared tachypneic, with moderate respiratory distress and hypoxia. Lung auscultation revealed clear lung sounds. Her vital signs upon arrival included a heart rate of 87 bpm, a blood pressure of 112/75, and hypoxia with an oxygen saturation level of 81%. An ECG (Figure 1) showed new ST elevation in V1 and V2 compared to a previous ECG (Figure 2), raising concern for a STEMI. Cardiology was consulted immediately, but due to the patient's history and vital signs, there was a high suspicion of PE. The patient underwent a CT angiography (CTA), revealing bilateral segmental pulmonary emboli with evidence of right heart strain (Figure 3). Laboratory results showed an elevated D-dimer of 2,191 ng/mL and Troponin I of 0.78 ng/mL. The patient was transferred to a tertiary care center for emergent thrombectomy.

Case 2

**Figure 4:**
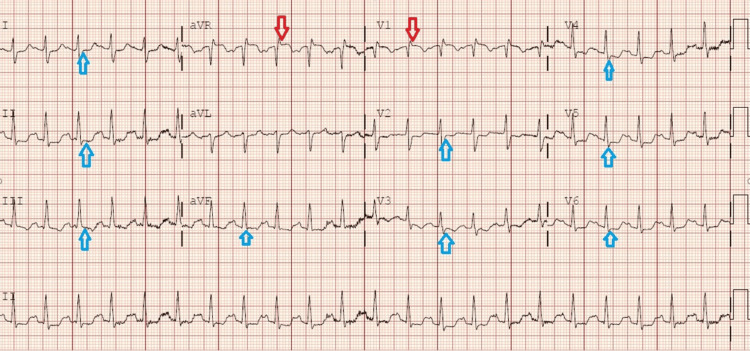
ECG on arrival to ED. Notable for ST elevation in AVR and V1 (red arrows) with diffuse ST depression (blue arrows) ECG, electrocardiogram

**Figure 5:**
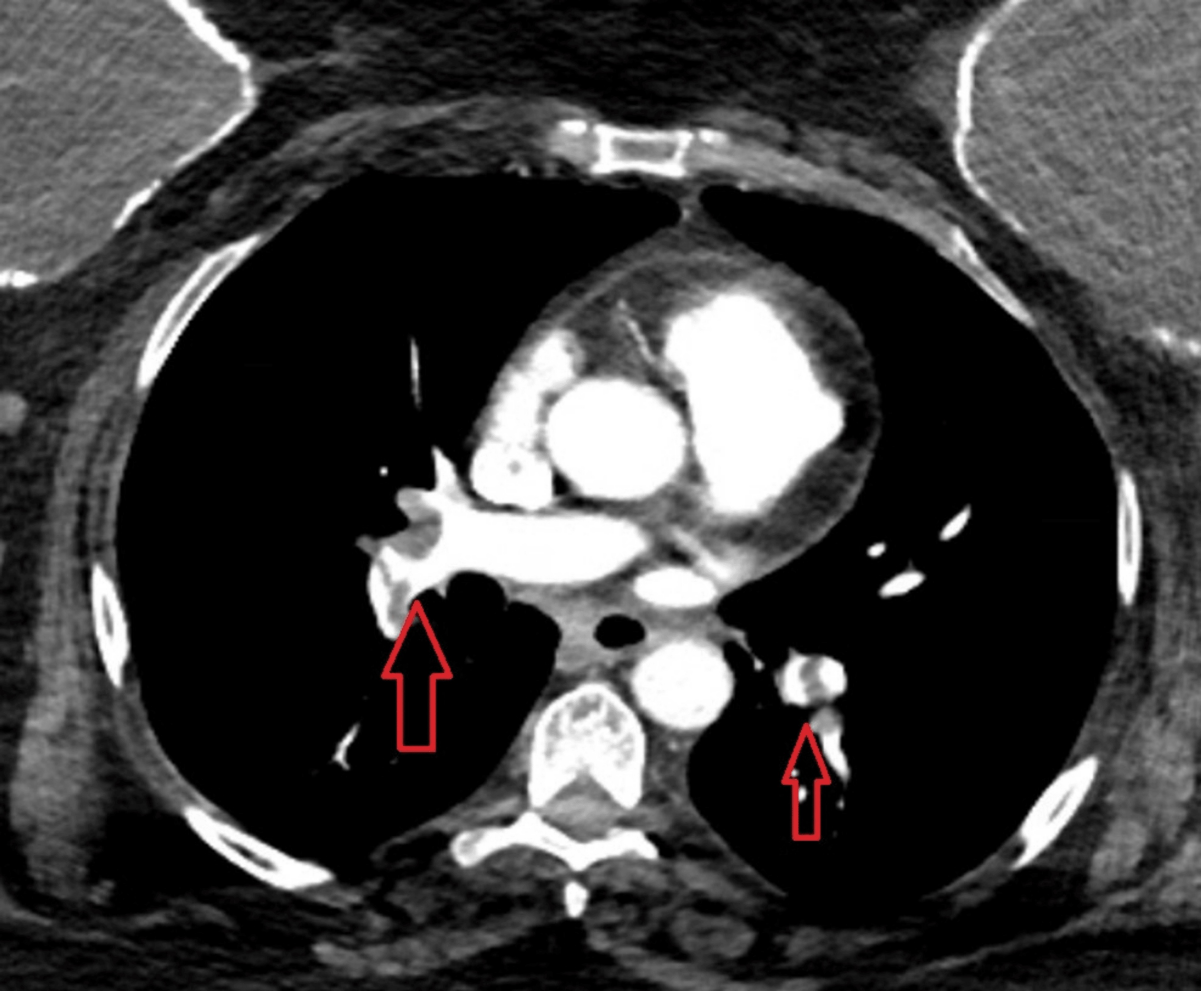
CT angiogram. Notable for bilateral pulmonary emboli (red arrows) with right heart strain

A 55-year-old female was brought in by EMS for chest pain and shortness of breath. She reported experiencing shortness of breath along with a pressure sensation in her chest that radiated to her back. Symptoms had been gradually worsening over the past three days but had become acutely worse a few hours prior to presentation. The patient had a past medical history of COPD and knee surgery two months prior. On examination, she was tachypneic and tachycardic, with moderate respiratory distress and hypoxia. Her lung sounds were clear. Her vital signs on arrival were a heart rate of 133 bpm, a blood pressure of 112/68, and a 98% oxygen saturation level of 8L nasal cannula. An ECG (Figure 4) revealed ST elevation in AVR and V1 with diffuse ST depression, concerning for an infarct with reciprocal changes. Due to the patient's high suspicion of PE, she was immediately sent to the CT scanner, which revealed bilateral pulmonary emboli with right heart strain (Figure 5). Laboratory results showed elevated troponin (67 ng/L), elevated BNP (990 pg/mL), and an elevated D-dimer (3596 ng/mL). The patient was emergently transferred to a tertiary care center for emergent thrombectomy.

## Discussion

Through these two case studies, we aim to provide a preliminary review of potential mechanisms underlying the phenomenon of PE presenting as STEMI, as well as shed light on the complexities of differential diagnosis and management in such scenarios. In the case of the 67-year-old female patient, the ECG on arrival showed new ST elevation in leads V1 and V2, which raised an immediate concern for STEMI. However, her history of knee surgery, recent prolonged immobility, and hypoxia warranted consideration of PE. The subsequent CTA revealed bilateral segmental pulmonary emboli with right heart strain, highlighting the need for clinicians to consider PE in differential diagnosis, even when initial ECG findings suggest STEMI. 

Similarly, the 55-year-old female patient presented with ST elevation in lead aVR and V1, along with diffuse ST depression, which could initially suggest a STEMI with reciprocal changes. Her history of COPD and recent surgery further increased the suspicion of PE. CTA confirmed bilateral pulmonary emboli with right heart strain. The elevated troponin and BNP levels also supported the differential diagnosis of right heart strain secondary to PE. PE obstructs the pulmonary arteries, which leads to increased pulmonary vascular resistance and subsequent myocardial shear stress on the right ventricle. This results in acute right ventricular dilatation and myocardial ischemia, which leads to the rise of BNP and troponin respectively [11]. This case reinforces the significance of considering PE in patients with atypical STEMI presentations, particularly in the context of risk factors for VTE.

The phenomenon of PE presenting as STEMI on ECG can be attributed to several potential mechanisms. One proposed mechanism is acute right ventricular strain due to the sudden increase in pulmonary vascular resistance, which can cause right ventricular ischemia and subsequent ECG changes that mimic anterior myocardial infarction [7]. The right ventricular strain leads to ST-segment elevation in the right precordial leads, as seen in both case reports, which can be misinterpreted as a STEMI.

Another mechanism is the presence of pre-existing cardiovascular conditions that may predispose patients to both PE and myocardial ischemia. For instance, hypoxia and hypotension associated with massive PE can exacerbate myocardial oxygen demand, leading to subendocardial ischemia and ECG changes characteristic of STEMI [12]. Additionally, elevated levels of cardiac biomarkers such as troponin and BNP, which are common in both PE and myocardial infarction, can further complicate the clinical picture and lead to diagnostic dilemmas.

A potential factor that we speculate may also contribute to explaining the overlapping EKG findings between both PEs and STEMIs may involve the protein Connexin-43 (Cx43) [13]. Cx43 plays a crucial role in cardiac conduction and its dysregulation can lead to significant arrhythmogenesis, as evidenced in conditions such as Duchenne muscular dystrophy (DMD). In DMD, Cx43 is pathologically mislocalized to the lateral sides of cardiomyocytes, rather than being confined to the intercalated discs where it normally functions to maintain proper ion trafficking and electrical signal propagation [13]. This mislocalization, along with increased overall Cx43 protein levels observed in both mouse models of DMD and human DMD tissues, contributes to the development of life-threatening arrhythmias. The aberrant Cx43 distribution can form nonfunctional hemichannels that disrupt the electrochemical gradient, leading to irregular cardiac conduction and increased susceptibility to arrhythmias [13]. In the context of the two cases presented, the altered ECG findings might be attributed to similar mechanisms of Cx43 dysregulation. Hypoxia and increased right ventricular pressure may similarly induce Cx43 lateralization and upregulation, contributing to electrical heterogeneity and mimicking STEMI-like ECG findings [14]. These alterations in Cx43 distribution can lead to the development of arrhythmias by impairing proper electrical conduction and promoting the formation of reentrant circuits [14,15]. The resultant ECG abnormalities, including ST-segment elevations in the right precordial leads, complicate the differential diagnosis between PE and STEMI, highlighting the need for advanced imaging techniques like CTPA for accurate diagnosis. 

Regardless of the potential mechanism, it is evident that distinguishing between PE and STEMI based on ECG findings alone can be highly challenging and often misleading. The overlap in clinical presentations and diagnostic markers necessitates a comprehensive and multifaceted approach to diagnosis and management. These case studies emphasize the critical role of a thorough clinical evaluation, including the consideration of patient history and risk factors, in guiding the diagnostic process. Factors such as recent surgery, immobility, and pre-existing conditions should prompt clinicians to maintain a high index of suspicion for PE, even when ECG findings might suggest a myocardial infarction. Additionally, the importance of advanced diagnostic imaging, such as CTA, cannot be overstated. This imaging modality not only helps in confirming the presence of PE but also provides vital information regarding right heart strain and other complications. In both cases, CTA was instrumental in establishing the correct diagnosis, thereby facilitating appropriate and timely treatment.

These cases also reinforce the need for ongoing research into the pathophysiological mechanisms underlying the ECG manifestations of PE. A deeper understanding of how conditions like hypoxia and right ventricular strain affect cardiac electrophysiology could lead to improved diagnostic criteria and novel therapeutic strategies. Exploring the role of proteins such as Cx43 in this context offers a promising avenue for future investigation. In clinical practice, these findings advocate for a more integrated approach to patient care, where clinical, biochemical, and imaging data are synthesized to form a cohesive diagnostic picture. Educating healthcare providers about the potential for PE to mimic STEMI on ECG is crucial in preventing misdiagnosis and ensuring that patients receive the most appropriate and effective treatment.

## Conclusions

The presented case studies illustrated the significant challenge of differentiating between PE and STEMI based on ECG findings alone, demonstrating the need for a comprehensive diagnostic approach that incorporates clinical history, risk factors, and advanced imaging techniques. We recommend the use of CTA for definitive diagnosis and cases where PE and STEMI symptoms overlap. Both cases highlight the potential for PE to present with ECG changes that closely mimic those of STEMI, thereby necessitating a high index of suspicion and the use of CTA to confirm the diagnosis. Understanding the underlying pathophysiological mechanisms, such as right ventricular strain and the role of Cx43 dysregulation, is crucial for improving diagnostic accuracy and treatment strategies. Ultimately, these cases advocate for greater clinical awareness and ongoing research to refine diagnostic criteria and enhance patient outcomes, ensuring that individuals with PE receive timely and appropriate care.
